# Draft Genome Sequences of Lactiplantibacillus plantarum Strains DSMZ 8862 and DSMZ 8866, Used as Feed Additives

**DOI:** 10.1128/mra.01166-21

**Published:** 2022-07-25

**Authors:** Bernd Pieper, Mareike Saathoff, Antje-Maria Lapschies, Torsten Semmler, Jutta Zielke, Marcus Fulde

**Affiliations:** a Dr. Pieper Technology and Product Development Ltd., Wuthenow, Germany; b Institute of Microbiology and Epizootics, Centre for Infection Medicine, Freie Universität Berlin, Berlin, Germany; c Genome Sequencing and Genomic Epidemiology, Robert Koch Institute, Berlin, Germany; University of Arizona

## Abstract

Here, we report the draft genome sequences of Lactiplantibacillus plantarum strains DSMZ 8862 and DSMZ 8866, which are currently being used as authorized feed additives in the European Union under regulation (EC) number 1831/2003. The draft genome sequences contain 3,334 kbp (DSMZ 8862) and 2,992 kbp (DSMZ 8866) in 15 and 8 contigs, respectively.

## ANNOUNCEMENT

Lactiplantibacillus plantarum is a member of the family *Lactobacillaceae* and colonizes many different environmental niches and hosts, including plants, fermented food, and the gastrointestinal tracts of various hosts (1). *L. plantarum* strains DSMZ 8862 and DSMZ 8866 were initially isolated from grass and maize environments, respectively, and have been used as a silage additive to improve the fermentation process during ensilage of various animal feedstuffs (2–4). In addition, both strains showed probiotic potential in pigs (5). We present here the draft genome sequences of these two strains in order to allow a better understanding of their efficacy and safety.

Both *L. plantarum* strains are part of the silage additive BIO-SIL (Dr. Pieper Technology and Product Development Ltd.). Single colonies of both strains were picked from Rogosa agar (Merck, Darmstadt, Germany) plates after growth at 30°C for 48 h. Prior to DNA isolation using the QIAamp DNA minikit (Hilden, Germany) for both Illumina and Nanopore sequencing, the strains were grown in brain heart infusion (BHI) broth (Difco) overnight at 37°C. Short-read sequencing was performed on an Illumina NextSeq sequencer using the v2.5 high-output reagent kit (Illumina Inc., San Diego, CA, USA) and the Nextera XT library preparation kit (Illumina), resulting in 150-bp paired-end reads and >90-fold coverage. Additionally, long-read sequencing was performed using the Oxford Nanopore MinION platform (Oxford, UK). MinION one-dimensional (1D) libraries were constructed using the SQK-RBK004 kit (Oxford Nanopore Technologies) and loaded according to the manufacturer’s instructions onto an R9.4 flow cell. The sequencing data were collected for 48 h. Guppy v4.0.11+f1071ce was used in high accuracy mode to call the Nanopore reads. A total amount of 1 ng extracted DNA was used as the starting material for sequencing with the Illumina NextSeq instrument and 400 ng for sequencing with the MinION instrument. A closed genome was generated by a *de novo* hybrid assembly using a combination of short and long reads with Unicycler v0.4.7 (6). A draft genome assembly created using SPAdes v3.12 (7) as part of the Unicycler pipeline and the consecutive connection of contigs using the long reads from MinION resulted for DSMZ 8862 in a single circular chromosomal DNA molecule of 3,334 kbp with a G+C content of 44.5% and 14 small contigs with sizes ranging from 1 to 62 kbp. For DSMZ 8866, the combined assembly resulted in a single circular chromosomal DNA molecule of 2,992 kbp with a G+C content of 45.5% and 7 small contigs with sizes ranging from 3 to 52 kbp.

Using ResFinder (8) and PlasmidFinder (9) through http://www.genomicepidemiology.org/ (accessed August 2019; default settings), neither resistance genes nor plasmids were identified. Furthermore, both *L. plantarum* strains were tested for susceptibility to antimicrobials in accordance with ISO 10932:2010 via a serial 2-fold dilution procedure in broth using antibiotic precoated microdilution plates (VetMIC Lact-1 and VetMIC Lact-2, National Veterinary Institute, Uppsala, Sweden). The tests were performed by two independent laboratories with similar results (Table 1). The current cutoff levels regarding the relevant antimicrobials for Lactiplantibacillus plantarum, when used as a feed additive, were not exceeded (10).

**TABLE 1:** Determination of the MICs of antibiotics for *L. plantarum* DSMZ 8862 and *L. plantarum* DSMZ 8866 according to ISO 10932:2010 using the VetMIC Lact-1 and Lact-2 ready-made microdilution plates^*a*^

	Antibiotic	MIC (mg/L)														Sum^*b*^	No growth or contaminated
		0.03	0.06	0.12	0.25	0.5	1	2	4	8	16	32	64	128	256		
*L. plantarum* DSMZ 8862	Gentamicin	–^*c*^	–	–	–	4	14	6	1	2						27	0
	Kanamycin	–	–	–	–	–	–				6	12	7	2		27	0
	Streptomycin	–	–	–	–					5	13	6	3			27	0
	Neomycin	–	–	–	–	1	5	16	3	2						27	0
	Tetracycline	–	–								8	19		–	–	27	0
	Erythromycin			9	10	6	2				–	–	–	–	–	27	0
	Clindamycin						3	17	7			–	–	–	–	27	0
	Chloramphenicol	–	–							27				–	–	27	0
	Ampicillin						3	9				–	–	–	–	12	0
	Penicillin								1	6	5	–	–	–	–	12	0
	Vancomycin	–	–	–										12^*d*^	–	12	0
	Quinupristin-dalfopristin							12			–	–	–	–	–	12	0
	Linezolid							3	9			–	–	–	–	12	0
	Trimethoprim	–	–		4	7	1							–	–	12	0
	Ciprofloxacin	–	–	–							2	9	1		–	12	0
	Rifampicin	–	–					3	5	3	1			–	–	12	0
*L. plantarum* DSMZ 8866	Gentamicin	–	–	–	–	4	12	15	5							36	0
	Kanamycin	–	–	–	–	–	–				6	17	10	3		36	0
	Streptomycin	–	–	–	–					2	22	8	4			36	0
	Neomycin	–	–	–	–		10	16	9		1					36	0
	Tetracycline	–	–								16	20		–	–	36	0
	Erythromycin			15	11	7	2	1			–	–	–	–	–	36	0
	Clindamycin						2	11	20	3		–	–	–	–	36	0
	Chloramphenicol	–	–						8	28				–	–	36	0
	Ampicillin					2	7	3				–	–	–	–	12	0
	Penicillin								1	2	9	–	–	–	–	12	0
	Vancomycin	–	–	–										12^*d*^	–	12	0
	Quinupristin-dalfopristin							12			–	–	–	–	–	12	0
	Linezolid							3	9			–	–	–	–	12	0
	Trimethoprim	–	–		12									–	–	12	0
	Ciprofloxacin	–	–	–							1	6	5		–	12	0
	Rifampicin	–	–				1	11						–	–	12	0

a The numbers in each row indicate the individual number of tests providing the respective results (MIC).

b Sum of independent tests.

c –, not determined.

d MIC (Minimal Inhibitory Concentration) > 128 mg/L.

### Data availability.

The complete genome sequences of Lactiplantibacillus plantarum DSMZ 8862 and DSMZ 8866 have been deposited at DDBJ/ENA/GenBank under the accession numbers JAHBMN000000000 and JAHHDW000000000, respectively. The raw reads have been deposited at NCBI under the BioProject accession number PRJNA728514 and the BioSample accession numbers SAMN26224025 (strain DSMZ 8862) and SAMN26224024 (strain DSMZ 8866).
